# Targeted inhibition of the Shroom3–Rho kinase protein–protein interaction circumvents Nogo66 to promote axon outgrowth

**DOI:** 10.1186/s12868-015-0171-5

**Published:** 2015-06-16

**Authors:** Heather M Dickson, Amanda Wilbur, Ashley A Reinke, Mathew A Young, Anne B Vojtek

**Affiliations:** Department of Biological Chemistry, University of Michigan Medical School, Ann Arbor, MI USA; Department of Pharmacology, University of Michigan Medical School, Ann Arbor, MI USA

**Keywords:** Neural regeneration, NogoA, Shroom3, ROCK, PirB, POSH, Protein–protein interaction inhibitors

## Abstract

**Background:**

Inhibitory molecules in the adult central nervous system, including NogoA, impede neural repair by blocking axon outgrowth. The actin-myosin regulatory protein Shroom3 directly interacts with Rho kinase and conveys axon outgrowth inhibitory signals from Nogo66, a C-terminal inhibitory domain of NogoA. The purpose of this study was to identify small molecules that block the Shroom3–Rho kinase protein–protein interaction as a means to modulate NogoA signaling and, in the longer term, enhance axon outgrowth during neural repair.

**Results:**

A high throughput screen for inhibitors of the Shroom3–Rho kinase protein–protein interaction identified CCG-17444 (Chem ID: 2816053). CCG-17444 inhibits the Shroom3–Rho kinase interaction in vitro with micromolar potency. This compound acts through an irreversible, covalent mechanism of action, targeting Shroom3 Cys1816 to inhibit the Shroom3–Rho kinase protein–protein interaction. Inhibition of the Shroom3–Rho kinase protein–protein interaction with CCG-17444 counteracts the inhibitory action of Nogo66 and enhances neurite outgrowth.

**Conclusions:**

This study identifies a small molecule inhibitor of the Shroom3–Rho kinase protein–protein interaction that circumvents the inhibitory action of Nogo66 in neurons. Identification of a small molecule compound that blocks the Shroom3–Rho kinase protein–protein interaction provides a first step towards a potential new strategy for enhancing neural repair.

## Background

The axon outgrowth inhibitory protein NogoA limits neural regeneration in the adult central nervous system [1–3]. Circumventing the action of NogoA with peptide antagonists, anti-NogoA antibodies, or soluble receptor fragments increases axon outgrowth and neural plasticity, resulting in improved functional recovery after stroke [4–7]. Behavioral recovery after stroke is also enhanced in knockout animals deficient in NogoA or receptors for NogoA [4, 8].

Nogo receptor 1 (NgR1) and paired immunoglobulin-like receptor B (PirB) are two distinct cell surface receptors that interact with Nogo66, a C-terminal 66 amino acid inhibitory domain of NogoA [9, 10]. NgR1 partners with LINGO-1 and p75 or TROY [11, 12]. Upon Nogo66 engagement of this receptor complex, calcium levels increase and RhoA and its effector Rho kinase are activated, leading to destabilization of the actin cytoskeleton and growth cone collapse [13]. Despite being structurally unrelated to NgR1, PirB also binds Nogo66 with high affinity, and Nogo66-PirB signaling inhibits neurite outgrowth [9, 14]. In addition to acting as a receptor for NogoA, PirB and its human ortholog leukocyte immunoglobulin-like receptor, subfamily B, member 2 (LILRB2) are receptors for beta amyloid; interaction of beta amyloid with PirB/LILRB2 impairs synaptic plasticity and enhances synaptic loss, suggesting a contribution to Alzheimer’s disease pathology [15].

PirB mediates Nogo66 neurite outgrowth inhibition through an intracellular signaling complex organized by the scaffold protein plenty of SH3s (POSH; also known as Sh3rf1) [14, 16]. This signaling complex includes the actin-myosin regulator Shroom3, Rho kinase, and leucine zipper kinase (LZK or MAP3K12) [14, 16]. Shroom3 directly binds to Rho kinase [16–19]. The Shroom3 interaction domain is located in a central coiled coil region of Rho kinase, directly upstream of the RhoA binding domain [18, 19]. Ectopic expression of this domain of Rho kinase disrupts the interaction of endogenous Shroom3 and Rho kinase and in neurons expression of this domain enhances neurite outgrowth [16, 19, 20]. This observation points to an important role for the Shroom3–Rho kinase protein–protein interaction in negatively regulating neurite outgrowth.

Here we report the identification and characterization of the first class of chemical inhibitors of the Shroom3–Rho kinase protein–protein interaction. A biochemical screen of 20,000 diverse chemical compounds identified nine potent inhibitors of the Shroom3–Rho kinase protein–protein interaction. Among these, CCG-17444 enhanced neurite outgrowth and circumvented Nogo66 inhibition in neurons. This compound binds covalently to Shroom3 and interferes with its ability to interact with Rho kinase. The identification of a small molecule compound that blocks the Shroom3–Rho kinase protein–protein interaction provides a first step towards a potential new strategy for neural repair.

## Results

### High throughput screen for Shroom3–Rho kinase inhibitors

**Figure 1:**
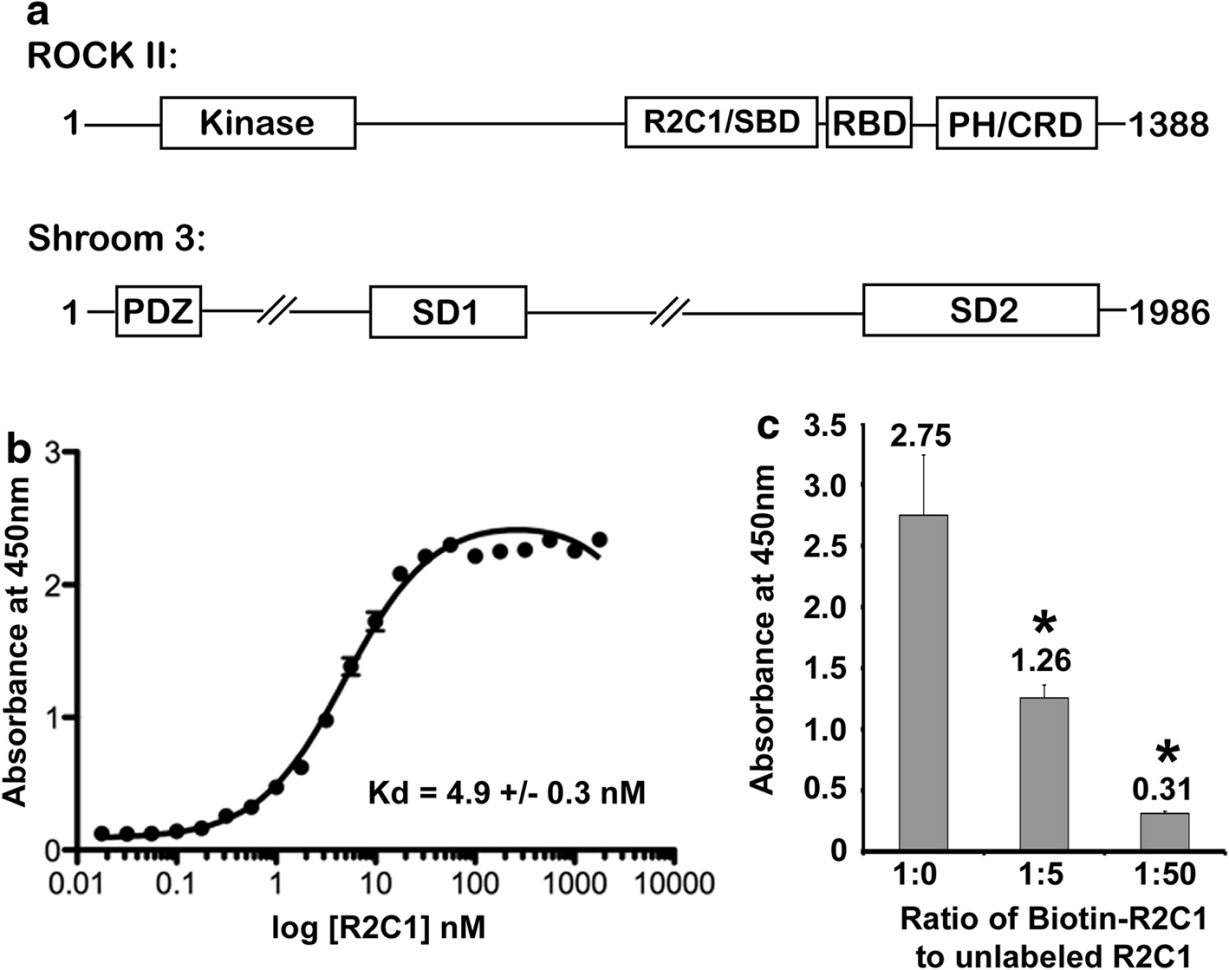
Shroom3 and Rho kinase interact with high affinity. **a** A schematic overview of the Shroom3 and ROCK II domain architecture. R2C1 is the Shroom binding domain (SBD) of ROCK II. Shroom domain (SD) 2 is the ROCK interacting domain of Shroom3. In these studies, R2C1 and SD2 encode amino acids 698–957 and 1,563–1,986 of ROCK II and Shroom3, respectively. **b** Shroom3 and ROCK II interact with nM affinity (Kd ± SEM, n = 8). **c** Unlabeled R2C1 competes with biotin labeled R2C1 for association with SD2 in a dose dependent manner (n = 4, *p < 0.05, Student’s *t* test).

Shroom3 and Rho kinase directly interact through the SD2 domain of Shroom3 and the R2-C1 domain of Rho kinase 2 (ROCK2) (Figure 1a). An enzyme linked immunosorbent assay (ELISA) platform was developed to detect the interaction of these two domains. Bacterially purified, unlabeled SD2 (aa 1,563–1,986) was directly immobilized on the surface of high binding 384 well microplates. R2-C1 (aa 698–957) was biotinylated. By varying the concentration of biotinylated R2-C1 with a fixed concentration of SD2, the apparent Kd was calculated to be 4.9 ± 0.3 nM (Kd ± SEM) (Figure 1b). To determine if binding of R2-C1 to SD2 was specific, a competition assay was performed. Unlabeled R2-C1 effectively competed with biotinylated R2-C1 for binding to immobilized SD2 (Figure 1c), demonstrating that the interaction assayed in the ELISA is due to specific binding of SD2 with R2-C1. The Z’ factor of the ELISA, a measure of assay dynamic range and variation, was 0.74, indicating the suitability of the SD2/R2-C1 ELISA platform for screening.

To identify inhibitors of the Shroom3/ROCK2 protein–protein interaction, 20,000 small molecule compounds were screened using the ELISA platform. Initial hits were defined as showing a signal that is greater than or equal to three standard deviations from the mean negative control per individual plate, e.g. greater than 20–30% inhibition (% effect). The primary screen of 20,000 compounds yielded 180 compounds for a 0.9% hit rate (Table 1).

**Table 1:** Summary of high throughput screening results

Total # compounds screened	20,000
Hits from primary screen	180 (0.9%)
Dose response	36 (0.18%)
Available for re-supply	32 (0.16%)
Confirmed inhibitors	27 (0.14%)
IC50 <30 μM	9 (0.05%)
Enhanced neurite outgrowth	1 (0.005%)

A 20,000 compound library was screened using the ELISA platform as described in Materials and Methods. 180 compounds were subject to dose response analysis. Of these 36 inhibited the Shroom3–ROCK interaction with pIC50 values greater than 4.0, had >60% efficacy at maximum dose tested, and had recovery rates in unrelated screens at <22%. 32 of the 36 chemicals were available for repurchase and of these 27 reconfirmed as inhibitors of the Shroom3–ROCK interaction. Nine compounds of the 27 confirmed hits have IC50 values less than 30 μM. One compound enhances neurite outgrowth.

Dose response analysis was performed with 180 hits from the primary screen. Compounds that titrated in dose response were triaged using % off-target effects, efficacy at maximum dose tested, and pIC50 values. By applying a stringent cutoff of greater than 60% inhibition in the ELISA and pIC50 values greater than 3.5, 36 candidate inhibitors of the Shroom3/ROCK2 protein–protein interaction were identified. 32 of the 36 were available for resupply.

**Figure 2:**
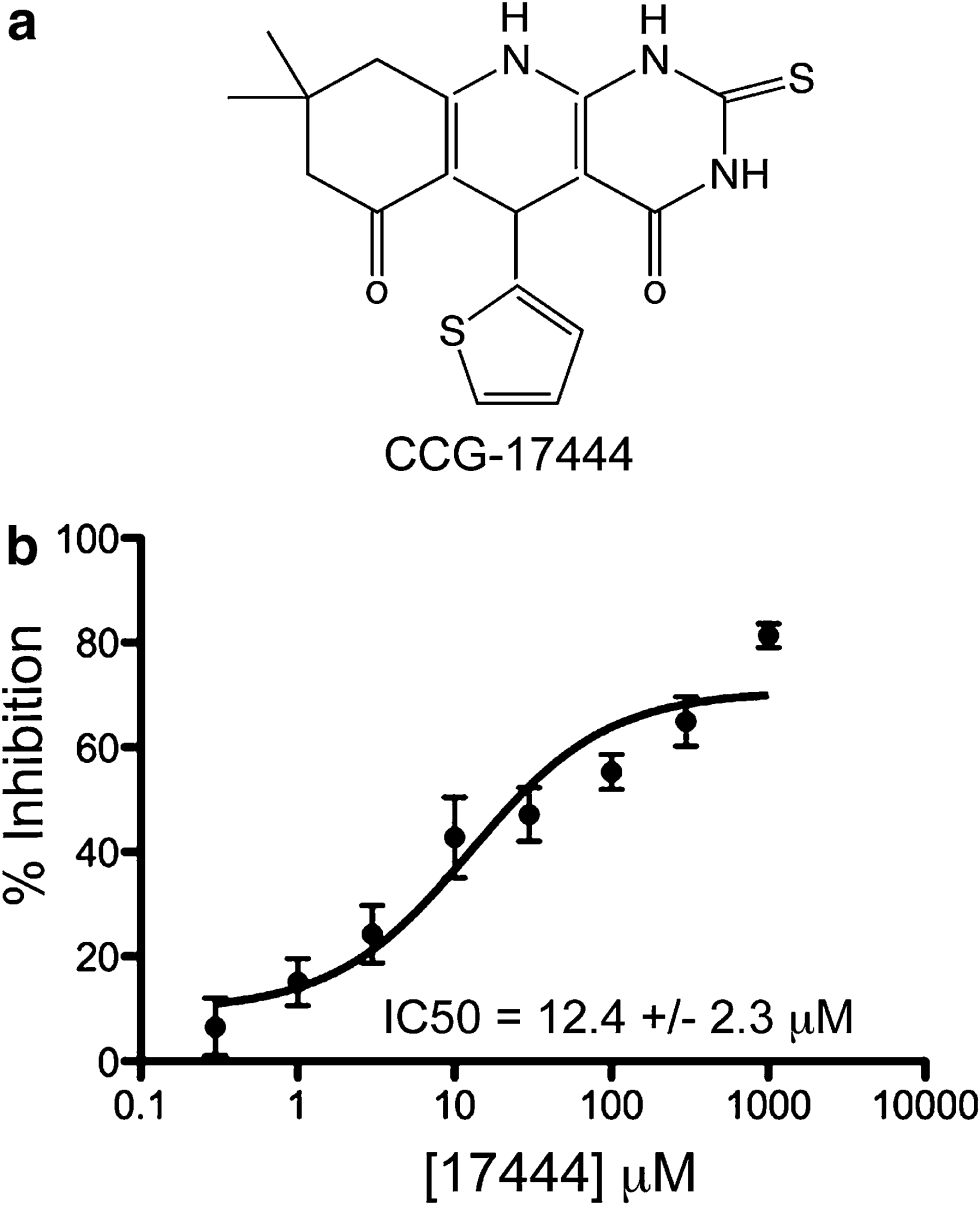
CCG-17444 inhibits the Shroom3–ROCK II protein–protein interaction. **a** Chemical structure of CCG-17444 (Chem ID: 2816053). **b** CCG-17444 inhibits the Shroom3–ROCK II interaction with an IC50 of 12.4 ± 2.3 (IC50 ± SEM, n = 3).

A follow-up dose response assay was performed using fresh powder samples. 27 compounds reconfirmed as hits and nine compounds had IC50 values of less than 30 μM. These nine compounds were tested for their ability to enhance neurite outgrowth in neurons, as hypothesized for an inhibitor of the NogoA signaling pathway. One compound, CCG-17444, enhanced neurite outgrowth (discussed below) and was defined as the primary hit from the screen (Figure 2a). CCG-17444 inhibited the Shroom3–ROCK interaction with an IC50 value of 12.4 ± 2.3 μM (IC50 ± SEM) (Figure 2b). To assess cytotoxicity, P19 neurons were treated with 25 μM CCG-17444 or DMSO vehicle control for 24 h and toxicity assessed using a resazurin-based assay that measures cellular reducing potential (Alamar blue). No increase in cytotoxicity was observed in CCG-17444 treated neurons relative to DMSO control treated neurons (data not shown).

### CCG-17444 enhances neurite outgrowth

**Figure 3:**
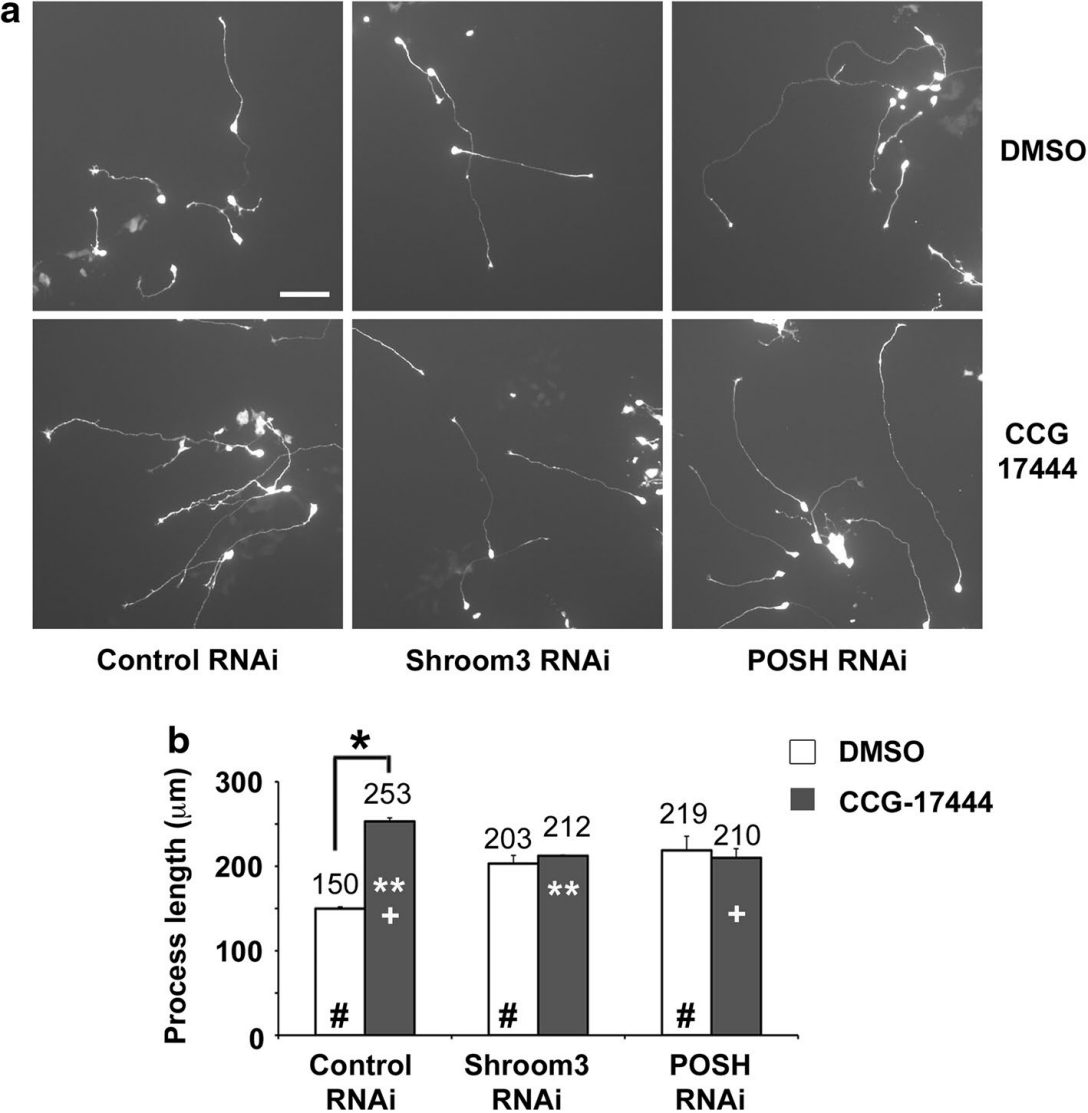
Inhibition of the Shroom3–ROCK protein–protein interaction with CCG-17444 enhances neurite outgrowth. P19-derived neurons were treated with 25 μM CCG-17444 or vehicle control (DMSO) for 24 h. **a**, **b** CCG-17444 enhances neurite length in neurons transfected with an RNAi control vector. Neurons transfected with POSH RNAi or Shroom3 RNAi vectors exhibit enhanced neurite length due to reduction of POSH or Shroom3 function but CCG-17444 does not further enhance neurite length, suggesting that CCG-17444 selectively targets the POSH/Shroom3 signaling complex (n = 3). Student’s t test: *p < 0.0001, Control RNAi vs CCG-17444 treated Control RNAi; ^#^p < 0.0001, Control RNAi vs Shroom3 or POSH RNAi; **p < 0.0005, CCG-17444 Control RNAi vs CCG-17444 Shroom3 RNAi; ^+^p < 0.0001, CCG-17444 Control RNAi vs CCG-17444 POSH RNAi. *Scale bar* 100 μm.

NogoA signals to the POSH/Shroom3/Rho kinase signaling complex to limit neurite outgrowth in cultured neurons [14]. Therefore, we hypothesized that pharmacological inhibition of the Shroom3/ROCK2 protein–protein interaction with CCG-17444 would relieve neurite outgrowth inhibition, as observed for RNA interference (RNAi) mediated reduction of POSH or Shroom3 function [14, 16]. To test this hypothesis, we examined the effect of compound treatment on neurite outgrowth in differentiated neurons derived from mouse P19 embryonic carcinoma cells [14, 16, 21, 22]. Neurons were generated by transfection with the neural basic helix-loop-helix protein Neurogenin 2 (Ngn2) [16, 21]. Control, Shroom3, or POSH RNAi vectors were co-transfected with Ngn2. 48 h after transfection, neurons were treated with vehicle control (DMSO) or 25 μM CCG-17444. 24 h later, neurons were fixed and stained for green fluorescent protein (GFP), which identifies the transfected neurons, and neurite outgrowth analyzed. P19-derived neurons treated with CCG-17444 exhibited an increase in neurite length relative to control treated neurons, similar to the increase observed for neurons with an RNAi-mediated decrease in POSH or Shroom3 (Figure 3a, b). P19-derived neurons treated with CCG-17444 at concentrations below 25 μM (e.g. 6.25 or 12.5 μM) did not exhibit an increase in neurite length relative to control DMSO treated neurons, and drug treatment at 50 μM increased neurite length to the same extent as 25 μM (data not shown).

To probe the mode of action of CCG-17444, we determined the effect of compound addition on neurite length for neurons expressing POSH or Shroom3 RNAi vectors. If the compound and RNAi operate on different signaling pathways to enhance neurite outgrowth, then compound addition and RNAi are expected to work additively or cooperatively to enhance neurite outgrowth. In contrast to control neurons, neurons expressing POSH or Shroom3 RNAi vectors did not exhibit an additional increase in neurite length upon CCG-17444 treatment (Figure 3b). This result suggests that the compound and RNAi operate on the same signaling pathway to regulate neurite length, supporting the hypothesis that CCG-17444 inhibits the Shroom3–Rho kinase signaling complex to limit neurite outgrowth.

### CCG-17444 is an irreversible inhibitor of the Shroom3–ROCK protein–protein interaction

**Figure 4:**
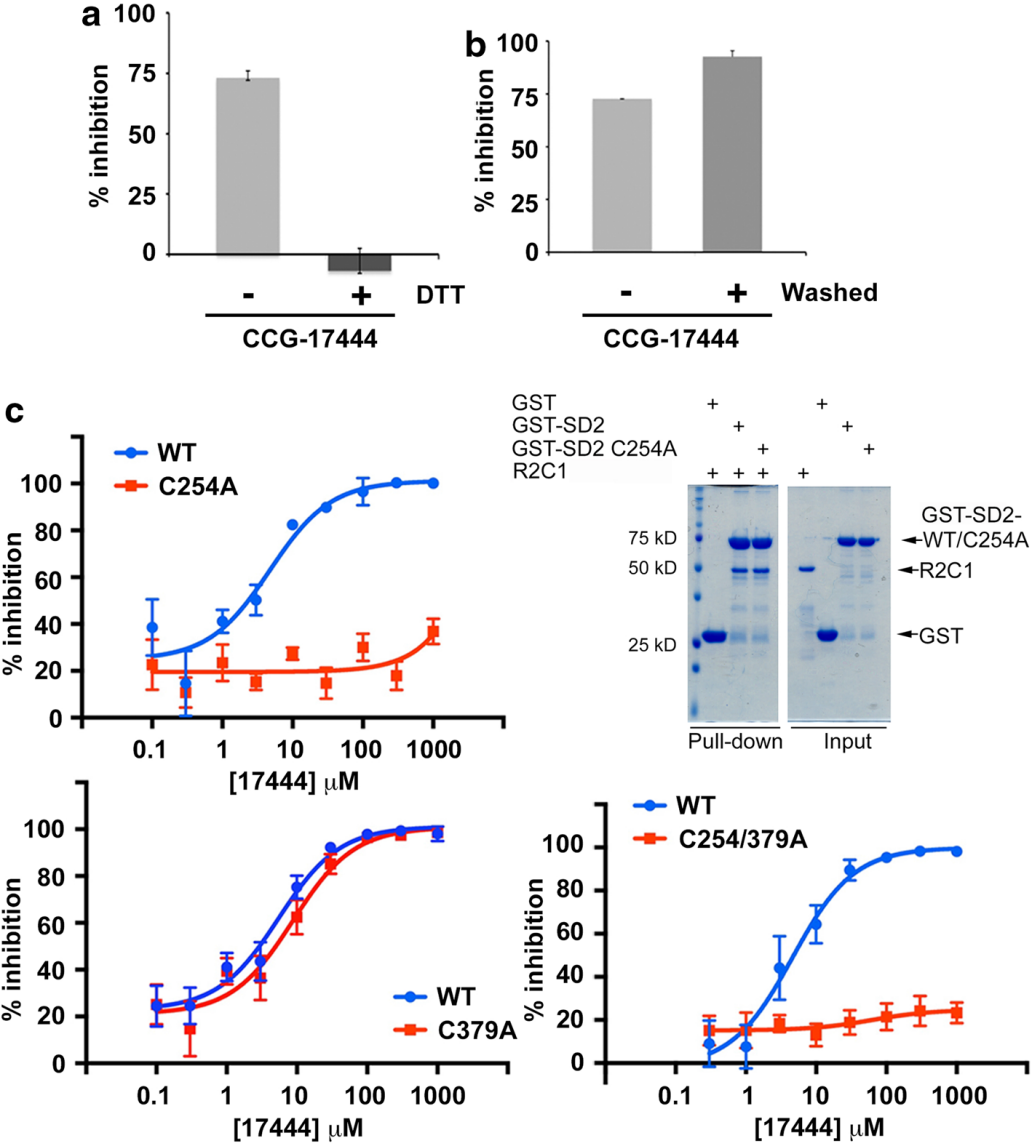
A single cysteine residue in the Shroom3 SD2 domain is required for CCG-17444 activity. **a** CCG-17444 activity is blocked by DTT. To investigate a thiol sensitive mechanism, DTT was added after plating SD2 and was present at all subsequent steps in the ELISA. (n = 3, p < 0.001, Student’s t test). **b** CCG-17444 is an irreversible inhibitor. After plating, SD2 was treated with CCG-17444 then wells were washed extensively to remove unbound compound prior to addition of R2-C1 (n = 3). **c** Cys254 of SD2 is the site of action of CCG-17444. CCG-17444 inhibits the interaction of WT SD2 with the ROCK R2-C1 domain but does not inhibit the interaction of SD2 Cys254Ala with ROCK (*upper left panel*). WT and Cys254Ala SD2 interact to similar extent with ROCK (*upper right panel*). CCG-17444 inhibits the interaction of SD2 Cys379Ala with R2-C1, suggesting that Cys379A is not a site of drug action (*lower left panel*). The double Cys to Ala mutant is indistinguishable from the single Cys254Ala mutant (*lower right panel*), indicating a single site of action of CCG-17444 (n = 3).

CCG-17444 (Figure 2a) could potentially function as a Michael acceptor; therefore, the activity of CCG-17444 in the presence of DTT was determined. The addition of DTT completely blocked the ability of CCG-17444 to inhibit the Shroom3–ROCK2 protein–protein interaction (Figure 4a), suggesting that CCG-17444 functions as a cysteine thiol reactive Michael acceptor, potentially modifying a sulfhydryl residue in one of the interacting partners. To test the reversibility of the interaction, CCG-17444 was incubated with the Shroom3 SD2 domain, the compound was then diluted and washed away prior to the addition of the ROCK R2-C1 domain. Diluting and washing away the compound had no effect on the ability of CCG-17444 to inhibit the protein–protein interaction (Figure 4b), indicating that CCG-17444 acts through an irreversible, covalent mechanism of action. In addition, this result identifies the Shroom3 SD2 domain as the target of the compound since the wash step occurs after incubation of compound with the Shroom3 SD2 domain and prior to addition of ROCK R2-C1.

To further examine the role of cysteine residues in the mechanism of action of CCG-17444, site directed mutagenesis was employed. The SD2 core domain (aa 1,762–1,952), which is sufficient to bind ROCK [18], contains two cysteine residues and each was mutated to alanine, individually or in combination. SD2 Cys 254 corresponds to Cys 1816 in full length mouse Shroom3 (accession number AAF13269); SD2 Cys379 corresponds to mouse Shroom3 Cys1941. CCG-17444 inhibited the ability of SD2 Cys379Ala to interact with R2-C1, indistinguishable from wild type (Figure 4c). In contrast, the interaction of SD2 Cys254Ala with R2-C1 was resistant to CCG-17444, identifying Cys254 in SD2 as the site of action of CCG-17444. Although resistant to drug inhibition, SD2 Cys254Ala bound to R2-C1 to a similar extent as SD2 wild type (Figure 4c). The interaction of the double mutant SD2 Cys254Ala Cys379Ala was indistinguishable from the single mutant Cys254Ala. These results identify SD2 Cys254 (mouse Shroom3 Cys1816) as the primary site of action of CCG-17444 and support the hypothesis that CCG-17444 interferes with the Shroom3–ROCK2 protein–protein interaction by forming a covalent adduct with cysteine 254 in the SD2 domain of Shroom3.

**Figure 5:**
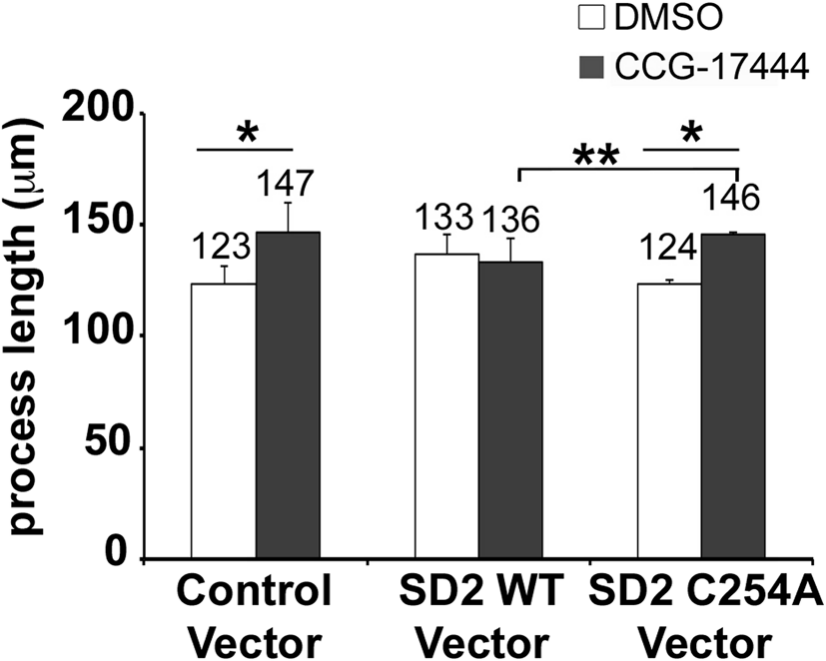
Overexpression of SD2 in neurons blocks CCG-17444 activity. P19-derived neurons were transfected with a control vector or SD2 expression vector (Shroom3 aa 1,762–1,986) and treated with DMSO (vehicle control) or 25 μM CCG-17444 for 24 h prior to analysis of neurite length. Ectopic expression of wild type but not Cys254Ala SD2 reverses the action of CCG-17444, demonstrating that the mechanism of action of CCG-17444 in neurons is inhibition of SD2 function (n = 3, *p < 0.0001, **p < 0.05 Student’s t test).

To further test the hypothesis that CCG-17444 regulates neurite outgrowth through its interaction with Shroom3, neurons overexpressing Shroom3 (aa 1,762–1,986), which includes the SD2 core domain, were treated with CCG-17444 and neurite outgrowth analyzed. We reasoned that overexpression of SD2, the target of the compound should titrate, or sequester, the compound away from the endogenous Shroom3–Rho kinase complex, preventing CCG-17444 from enhancing neurite outgrowth. Neurons transfected with a control vector exhibited an increase in neurite length when treated with CCG-17444 (Figure 5). DMSO treated neurons overexpressing SD2 (Shroom3 aa 1,762–1,986) exhibited a modest increase in neurite length relative to DMSO treated neurons transfected with a control vector, an increase from 123 ± 8 to 133 ± 11 μm (neurite length ± SD, p < 0.01). This increase may be due to this domain exerting a weak dominant negative effect, potentially by interfering with the interaction of endogenous Shroom3 and Rho kinase. Consistent with the hypothesis being tested, overexpression of SD2 reversed the action of CCG-17444, as neurite length of CCG-17444 treated SD2 expressing neurons was indistinguishable from DMSO treated SD2 expressing neurons. Notably, neurite outgrowth was increased in neurons overexpressing a mutant SD2 domain unable to bind CCG-17444, Cys254Ala. The observation that ectopic expression of wild type but not mutant SD2 reversed the action of CCG-17444 supports the hypothesis that CCG-17444 enhances neurite outgrowth by interfering with the function of the Shroom3 SD2 domain.

### CCG-17444 relieves neurite outgrowth inhibition by Nogo66

**Figure 6:**
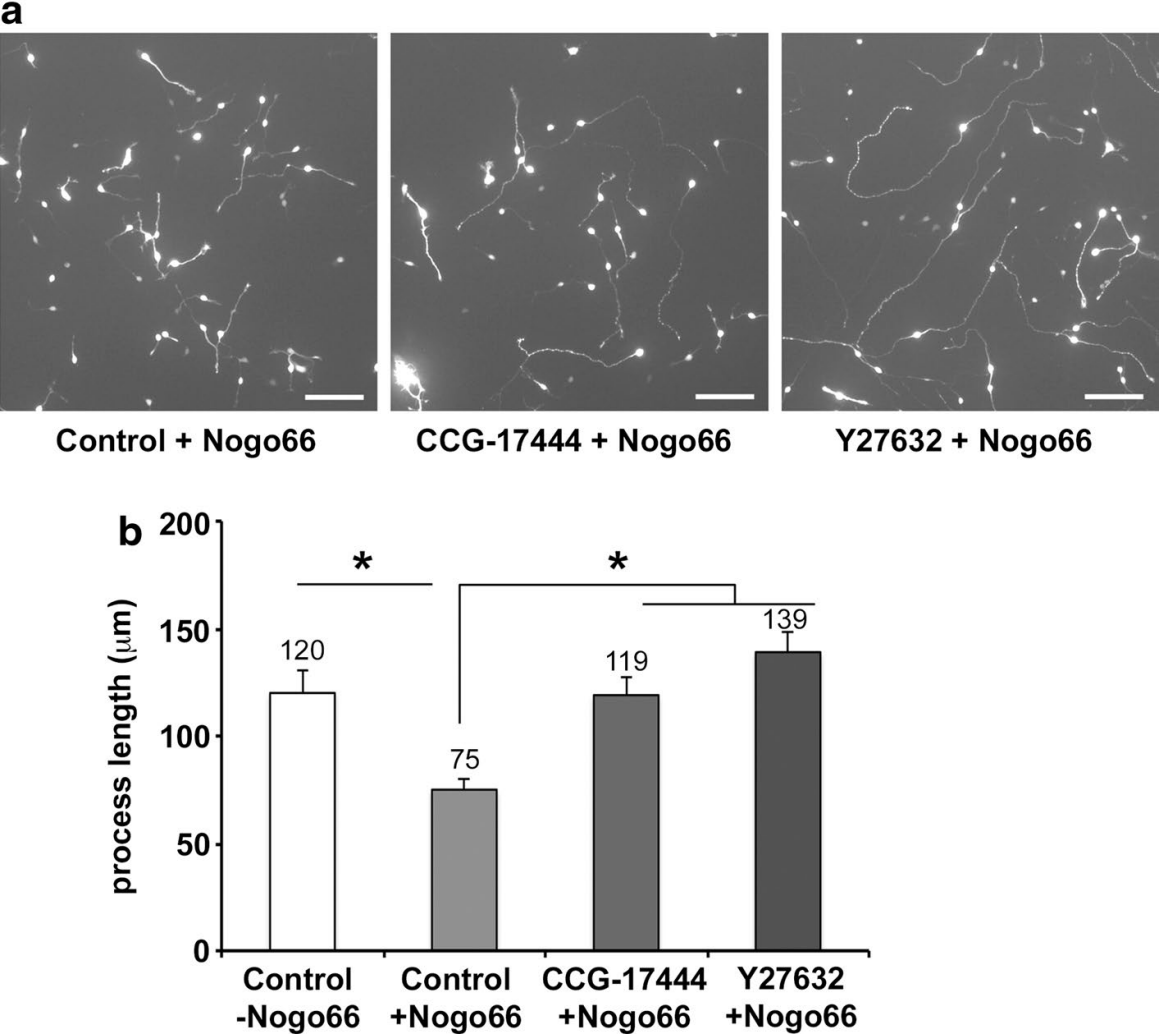
CCG-17444 enhances neurite outgrowth in CGNs and counteracts the inhibitory action of Nogo66. Postnatal d8 CGNs were treated for 24 h with CCG-17444, vehicle control, or the ROCK inhibitor Y27632 (positive control). **a** Nogo66 inhibits neurite outgrowth in CGNs, and CCG-17444 counteracts this inhibition. *Scale bar* 100 μm. **b** Quantitation of neurite length. Data are mean ± SD (n = 3, *p < 0.0001, Student’s t test).

Based on our published studies that demonstrated that Nogo66 signals through the PirB receptor to the POSH complex to limit neurite outgrowth [14], we hypothesized that chemical inhibitors that disrupt the Shroom3–ROCK protein–protein interaction in vitro would counteract the action of Nogo66 in neurons. To test this hypothesis, primary cerebellar granule neurons (CGNs) were prepared from postnatal day 8 mice and nucleofected with an expression vector for GFP to facilitate quantitative determination of neurite length of individual neurons. Nucleofected CGNs were plated to poly-l-lysine/laminin (control) or poly-l-lysine/laminin plus Nogo66. Six hours after nucleofection and plating, compounds were added and, 24 h later, neurons were fixed and processed for determination of neurite length. Neurons treated with CCG-17444 show enhanced neurite length on the inhibitory substrate Nogo66 relative to control neurons (Figure 6). Consistent with previous reports [23, 24], neurons treated with the Rho kinase inhibitor Y27632 (positive control) show an increase in neurite length relative to control neurons in the presence of Nogo66. Thus, pharmacological inhibition of the endogenous Shroom3–Rho kinase protein–protein interaction with CCG-17444 enables primary neurons to overcome growth mediated inhibition by Nogo66.

## Discussion

Employing a chemical screening methodology, we identified a small molecule inhibitor of the Shroom3–Rho kinase protein–protein interaction CCG-17444 that exhibits micromolar potency. The Shroom3–Rho kinase signaling complex, together with the POSH scaffold protein, relays Nogo66-mediated neurite outgrowth inhibition [14, 16]. Importantly, neurons treated with CCG-17444 are refractory to the inhibitory action of Nogo66. To our knowledge, this is the first identification of a small molecule chemical compound that relieves Nogo66 inhibition by disrupting a protein–protein interaction in an intracellular signal transduction complex.

CCG-17444 was identified on the basis of its ability to interfere with the interaction of Shroom3 and Rho kinase in vitro. Three observations indicate that the compound targets the Shroom3–Rho kinase signaling complex in vivo. First, genetic inhibition of the POSH/Shroom3/Rho kinase signaling pathway in neurons has a comparable outcome to CCG-17444 treatment. Namely, CCG-17444 relieves Nogo66 inhibition, as does RNAi-mediated reduction of POSH or Shroom3. Second, CCG-17444 fails to enhance neurite outgrowth in neurons deficient in either Shroom3 or POSH function, indicating that compound and RNAi target the same, not different, signaling pathways to enhance neurite outgrowth. Third, cellular overexpression of wild type but not Cys254 mutant SD2 core domain reverses the action of CCG-17444 on neurite outgrowth, consistent with the overexpressed SD2 domain acting as a sink to titrate the compound away from the endogenous Shroom3–Rho kinase complex. We conclude that CCG-17444 circumvents neurite outgrowth inhibition by Nogo66 by interfering with the POSH/Shroom3/ROCK signaling pathway.

The chemical structure of CCG-17444 suggests that it has potential to function as a Michael acceptor, undergoing a nucleophilic attack by a free sulfhydryl of a Michael donor cysteine. Consistent with this, inhibition of the Shroom3 SD2/Rho kinase R2-C1 protein–protein interaction by CCG-17444 is blocked by the addition of DTT and is irreversible. Moreover, CCG-17444 does not inhibit the interaction between SD2 Cys254Ala and R2-C1, identifying Cys254 of SD2 (Cys 1816 of mouse Shroom3) as the site of action of the compound. On the basis of these observations, CCG-17444 is proposed to function by a covalent mechanism, with the compound binding to Cys254 of the SD2 domain of Shroom3 to inhibit the interaction between Shroom3 and Rho kinase.

In vertebrates, two additional Shroom family members, Shroom2 and Shroom4, have been identified. Shroom proteins regulate cytoskeletal architecture and have important but distinct roles in development. Shroom3 regulates neural tube closure whereas Shroom2 regulates morphogenesis of the vasculature [25, 26]. In addition, Shroom2 regulates egg pigmentation in amphibians and melanosome biogenesis and pigmentation in the Xenopus eye [27, 28]. Mutations in Shroom4 have been linked to intellectual disability [29–32]. Shroom proteins have a conserved SD2 domain and interact with Rho kinase to regulate cell shape and tissue architecture [25, 33, 34]. The cysteine targeted by CCG-17444 in Shroom3 is also present in Shroom2 and Shroom4. However, addition of CCG-17444 has no further effect on the Shroom3 RNAi phenotype (Figure 3), suggesting that Shroom2 and/or Shroom4, if expressed in P19-derived neurons, are not playing a significant role in Nogo66 regulated neurite outgrowth inhibition. Whether CCG-17444 inhibits the interaction of Shroom2 and Shroom4 with ROCK in other contexts in vitro or in vivo remains to be determined.

The SD2 domain of Drosophila Shroom (dShrm) is a three-segmented coiled-coil dimer with internal symmetry [18]. Cys254 in SD2, conserved in mouse (accession number: AAF13269) and human, is equivalent to dShrm Val1446, which is located at the bend linking HelixA to HelixB. Based on the model proposed by Mohan et al. [18] that the SD2 dimer undergoes a conformational change upon binding to Rho kinase centered on the inflection point of internal symmetry, we propose that CCG-17444 blocks the interaction of Shroom3 and Rho kinase by an allosteric inhibitory mechanism.

Protein–protein interactions are prevalent and central to many biological processes but present unique challenges for probe and drug discovery efforts, including large, relatively flat surface areas and low affinity [35, 36]. Despite presenting unique challenges, there has been an increase in the discovery of small molecules that target protein–protein interactions. A large fraction of these target protein–protein interactions with tight affinities (<1 μM) and small surface areas [37]. Consistent with this paradigm, Shroom3 and Rho kinase interact with a relatively tight affinity (4.9 nM for mouse Shroom3/Rho kinase II); however, CCG-17444 interacts with a specific Cys residue, potentially blocking an allosteric change, so small surface area across the protein–protein interface does not appear to be a major determinant in this case. Thus, small molecule compounds are likely to block protein–protein interactions through diverse modes of action.

NogoA/Nogo66, EphrinA5, and chondroitin sulfate proteoglycans converge on the RhoA/Rho kinase signaling pathway and actively prevent axonal growth after injury [2, 3, 38, 39]. Blockade of these axon outgrowth inhibitors with antibodies that block receptor function, competitive peptide antagonists, or soluble receptor decoys enhances functional recovery after CNS injury [4–7, 39]. Distinct from strategies that block receptor function, CCG-17444 inhibits a critical protein–protein interaction within a key intracellular signaling hub that regulates neurite outgrowth inhibition, the interaction between Shroom3 and Rho kinase. Rho kinase regulates a diverse array of biological outcomes [40–42] so precisely blocking Rho kinase activity in a signaling complex dedicated to regulating neurite outgrowth inhibition, rather than all Rho kinase enzymatic activity, is likely to be more specific and less prone to off-target effects. A significant contributor to disability after CNS injury is the failure of the adult CNS to undergo neural repair. Blocking protein–protein interactions within specific signaling hubs may provide new avenues for enabling neural repair in the damaged CNS.

## Conclusions

We identified the first small molecule inhibitor of the Shroom3–Rho kinase protein–protein interaction. Blocking the interaction of Shroom3 with Rho kinase in neurons with CCG-17444 circumvents Nogo66 inhibition in neurons. This study demonstrates the feasibility of targeting an intracellular signaling hub as well as protein–protein interactions to counteract Nogo66 signaling, indicating new directions for neural repair strategies.

## Methods

### Recombinant proteins

GST-SD2, a GST fusion to the mouse Shroom3 SD2 domain (aa 1,563–1,986) expressed in pGST-SD2, and HisSUMO-R2C1, a His-Sumo epitope fusion to mouse ROCK R2C1 (aa 698–957) were produced in *Escherichia coli*. Briefly, *E. coli* were lysed by sonication in PBS+ buffer (GST purification: PBS with 0.1 mM phenylmethylsulfonyl fluoride, 14 µg/mL aprotinin, 0.1% β-mercaptoethanol, 1 µM leupeptin, 1 µM pepstatin) (His purification: PBS with 0.1 mM phenylmethylsulfonyl fluoride, 14 µg/mL aprotinin, 0.1% β-mercaptoethanol, 1 µM leupeptin, 1 µM pepstatin, 25 mM imidazole). Triton X-100 was added to the lysate at 1% of the final volume. Lysates were incubated with prewashed glutathione agarose or HisPur Ni-NTA resin (Thermo Scientific) for 1 h at 25°C. Purified protein was eluted with GST elution buffer (50 mM Tris buffer with 100 mM reduced glutathione, pH 8) or His elution buffer (PBS+ with 250 mM Imidazole). HisSUMO-R2C1 was dialyzed overnight at 4°C in PBS and stored in 25% glycerol at −20°C. GST-SD2 was dialyzed for 3 h at 4°C in PBS with three buffer changes. The GST epitope tag was removed using His-TEV (S219V)-Arg Protease overnight at a concentration of 1 µg TEV protease per 100 µg of GST-SD2. TEV protease and free GST was removed from purified SD2 by incubation overnight at 4°C with prewashed glutathione agarose and HisPur Ni-NTA resin. SD2 was stored at −20°C in 25% glycerol.

HisSUMO-R2C1 was biotinylated (NHS-PEO_4_-Biotinylation Kit, Pierce). Briefly, biotinylation reactions were carried out at a 20:1 molar ratio of NHS-PEO_4_-biotin to HisSUMO-R2C1 in PBS (pH 7.4). HisSUMO-R2C1 was labeled for 2 h at 4°C. After the incubation, the unreacted NHS-PEO_4_-biotin was removed with buffer exchange in PBS (pH 7.4) using Zeba Desalt Spin Columns (2 mL, MWCO = 7,000 Da) (Pierce). The average extent of labeling for HisSUMO-R2C1 was estimated to be four biotin molecules per 1 mol of protein using the HABA assay, a measurement of the extent of biotinylation, as per the manufacturer’s protocol. Biotin-R2C1 was stored at −20°C in 25% glycerol.

For the pull-down assay in Figure 4c, wild type or mutant GST-SD2 (5 μg), bound to glutathione agarose resin, was incubated overnight with His-Sumo-R2-C1 (12.5 μg). Pull down samples were washed 3× in Triton IP buffer and 1× in PBS prior to SDS-PAGE. Proteins were detected by Coomassie G-250 (Gel Code Blue, Pierce). 1 μg of SD2 or R2-C1 was included as a gel loading (input) control.

### Affinity determination and competition assays

Apparent binding affinity (K_d_) was determined by immobilizing 0.5 µg of SD2 diluted in 75 µL PBS for 16 h at 4°C on 96-well Immulon 2B high binding plates (Thermo Scientific). Plates were blocked for 1 h at 25°C in SuperBlock T20 (TBS) Blocking buffer (Thermo Scientific). Concentrations of Biotin-R2C1 diluted in TBS-1 (20 mM Tris HCl, 150 mM KCl, 0.5% Triton X-100, pH 7.9) with 0.5% bovine serum albumin (BSA) were added from 0 to 1,778 nM for a total of 11 concentration points for 1 h at 25°C. Unbound protein was removed with four washes in TBS-2 (20 mM Tris HCl, 300 mM KCl, 0.5% Triton X-100, pH 7.9). High Sensitive NeutrAvidin-HRP was added at a dilution of 1:40,000 in TBS-3 (25 mM Tris HCl, 8.25 mM Tris Base, 154 mM NaCl, 2% BSA, 0.05% Tween-20) for 1 h at 25°C. Excess SUMO antibody was removed with 4 washes in TBS-T (25 mM Tris HCl, 137 mM NaCl, 2.7 mM KCl, 0.1% Tween-20). TMB substrate (Pierce) was added for 15 min and quenched with 0.18 M H_2_SO_4_. Absorbance was measured at 450 nm using a SpectraMax M5 microplate reader. The K_d_ was calculated using GraphPad Prism 4.0 using a hyperbolic fit with a non-zero intercept (∆A = ∆A_max_*[R2C1]/(K_d_ + [R2C1]). ∆A = absorbance change; ∆A_max_ = maximum absorbance change; [R2C1] = R2C1 concentration. For competition ELISAs, 100 ng of Biotin-R2C1 was incubated for 1 h at 25°C with unlabeled R2C1 (1–10 µg), and processed as described above.

### High-throughput screening

#### Primary screen

Initial assay development was performed in 96-well Immulon 2B high-binding plates (Thermo Scientific). The assay was then optimized for high-throughput screening in 384-well plates. 20,000 compounds (ChemDiv) were screened in the Center for Chemical Genomics at the University of Michigan. All reagent additions were performed using Thermo Labsystems Multidrop, and plate washes were performed using Bio Tek EL406 washer/aspirator. 150 ng of SD2 diluted in PBS (pH, 7.4) was immobilized for 16 h at 4°C on 384-well high-binding plates (Perkin Elmer). Unbound protein was removed with two washes of PBS (pH 7.4). Plates were blocked for 1 h at 25°C in SuperBlock T20 (TBS) Blocking buffer (Thermo Scientific), followed by two washes with PBS (pH 7.4). 20 µL of buffer A (20 mM Tris HCl, 150 mM KCl, 0.05% Triton X-100, 0.5% BSA, pH 7.9) was added to all wells. 200 nL of compounds were pin-tooled (one per well) into columns 3–22 resulting in a final concentration of 10 µM using the Biomex FX (Beckman). 200 nL of DMSO was added to control columns 1–2 (negative control) and 23–24 (positive control). 5 µg of unlabeled R2C1 in 20 µL of buffer A was added to columns 23–24 as a positive control for inhibition. After 30 min at 25°C, 10 ng of Biotin-R2C1 in 10 µL of buffer A was added to all wells and incubated for 1 h at 25°C. Unbound protein was removed with three washes (buffer A supplemented with 300 mM KCl and 0.5% Triton X-100). 40 µL of 1:40,000 NeutrAvidin-HRP diluted in TBS-3 was added to all wells and incubated for 45 min at 25°C. Plates were washed three times in TBS-T, followed by the addition of 20 µL of TMB substrate for 5 min. The TMB reaction was quenched with 20 µL 0.18 M H_2_SO_4_. Absorbance was measured at 450 nm using an automated PHERAstar plate reader (BMG Labs). Hits were defined as percent inhibition greater than 3 standard deviations (3 SD) from the mean of the negative control for inhibition (159 actives). Additional active samples based on percent effect values and standard deviation values were also analyzed (6 and 15 samples, respectively).

#### Dose response and hit selection criteria

Dose–response confirmation (180 compounds) was performed following the ELISA screening platform. Compound dilutions of 100, 59.8, 35.9, 21.5, 12.9, 7.69, 4.61, and 2.70 µM were delivered using the Mosquito X1 (TTP Labtech) in duplicate. Compounds with at least 30% inhibition and a pIC50 of 3.5 were considered active (74 compounds). Compounds with greater than 22% promiscuity and less than 60% efficacy were removed. The application of these selection criteria resulted in 36 compounds. IC_50_ values were calculated with GraphPad Prism 4.0 using nonlinear regression and the log (inhibitor) vs. response, variable slope equation. Clustering was performed using DataMiner by the Tripos algorithm OptiSim under the criteria of 65% or greater similarity.

#### Dose response with fresh powder

32 of the 36 hits from the primary screen were available for purchase from ChemDiv and were diluted in DMSO and stored at −20°C. 150 ng of SD2 diluted in 25 μL PBS was immobilized for 16 h at 4°C on 384-well high-binding plates (Perkin Elmer). Following blocking, serial compound dilutions of 1,000, 300, 100, 30, 10, 3, 1, and 0.3 μM were delivered in triplicate and incubated for 30 min at 25°C. 10 ng of biotin-R2C1 was added (1.5 ng in Figure 4) and incubated for 1 h at 25°C then the ELISA was completed as described in primary screen. 27 of the 32 compounds were confirmed as active hits. Compounds with an IC_50_ of less than 30 μM were prioritized (nine compounds). The IC50 values were calculated using GraphPad Prism with nonlinear regression and the log (inhibitor) vs. response, variable slope equation.

#### DTT and reversibility experiments

After plating 150 ng SD2, 1 mM DTT was added and was present throughout the assay. CCG-17444 was added at a concentration of 75 μM prior to addition of 1.5 ng biotin-R2-C1. To test reversibility, wells were washed five times for 2 min each with buffer A after a 30-min incubation of 75 μM CCG-17444 with SD2, prior to addition of biotin R2-C1. Biotinylated R2-C1 was detected with neutravidin HRP as described above. Absorbance was measured at 450 nm using a Tecan safire^2^ microplate reader.

### Neurite outgrowth experiments

#### P19-derived neurons

P19 neurons were generated by transfection of pluripotent P19 embryonal carcinoma cells with the neural basic helix-loop-helix transcription factor Ngn2. P-19 derived neurons express neuronal markers, adopt a neuronal morphology, and are electrically excitable [21]. A GFP expression vector is included in the transfection and readily enables the identification of the transfected cells. GFP is present both in the cell body and in neurites. For neurite length measurements, the length of the longest neurite per cell (ca. 3× cell body or 50 μm) was measured; GFP positive neurites are positive for the axon marker Tau [16] and the neuron specific tubulin TuJ1 [21, 22]. P19 cells, grown in minimal essential medium-α supplemented with 7.5% calf serum, 2.5% fetal bovine serum, and penicillin–streptomycin were plated the day before transfection to a density of 2 × 10^5^ cells/well of a 12-well dish and transfected the next day with 2.5 µg of total DNA (0.75 µg of Ngn2, 0.75 µg of GFP, and 1 µg of pUI4/UI5 RNAi expression vectors). In Figure 5, 1.25 µg of control or an SD2 expression vector (SD2 amino acids 1,768–1,986) was included with Ngn and GFP. RNAi vectors targeting POSH and Shroom3, as well as control luciferase RNAi vectors, have been described previously (CS2 Vector Resource, RRID:nif-0000-3027) [14, 16, 43, 44]. 4–6 h after transfection, cells were re-split 1:5 or 1:6 to laminin-coated dishes (2 μg/ml, Invitrogen). 18–20 h later, the cells were transferred into Opti-mem supplemented with 1% FBS and penicillin–streptomycin. CCG-17444 was added 48 h after transfection at final concentration of 25 μM (Figures 3, 5). 72 h after transfection, cells were fixed in 3.7% formaldehyde in PBS and stained for GFP to identify transfected cells [14, 16, 21]. Similar results are obtained when neurite length is determined by staining for GFP or neuron specific markers.

#### Cerebellar granule neurons

CGNs were isolated and plated in the presence or absence of Nogo66 and neurite length determined 30 h post-plating, as described [9, 14, 45, 46]. All care and procedures for mice were performed in accordance with the guidelines and approval of the University of Michigan University Committee on Use and Care of Animals. 12-well dishes (Corning) were coated with 10 μg/ml poly-l-lysine for 4 h then overnight with 2 μg/ml laminin at room temperature, or laminin plus bacterially expressed His-SUMO Nogo66 (0.75 μg/cm^2^) at 4°C. Postnatal d8 cerebellar granule neurons (CGNs) were nucleofected as described previously with a total of 6 μg DNA [14]. 6 h post nucleofection, Y-27632 (10 μM, Calbiochem, San Diego, CA, USA), a ROCK 1/2 inhibitor, or CCG-17444 (25 μM of 3347-0032, ChemDiv, San Diego, CA, USA), were added to CGNs in culture media [DMEM (Invitrogen) supplemented with 2% B27 (Invitrogen), and 1% penicillin/streptomycin (Invitrogen)]. Cells were fixed in 3.7% formaldehyde in PBS 24 h post inhibitor addition. Cells were stained with anti-GFP primary antibody [Molecular Probes (Invitrogen) Cat# A11122 RRID:AB_221569] followed by detection with Alexa Fluor 488 goat anti-rabbit (Life Technologies Cat# A11034 RRID:AB_10562715).

#### Measurement of neurite length

Photographs of GFP-positive neurons were imaged with a digital camera; the length of the longest neurite per cell (50 μm or greater, ~3× the cell body) was measured using the polyline function in MicroSuite Special Edition imaging software. For assessment of inhibition by Nogo66, neurites less than 50 μm were also measured. At least 100 neurons per condition per experiment were quantitated for neurite length in three independent experiments.

## Abbreviations

NgR1: Nogo receptor 1
PirB: paired immunoglobulin like receptor
LILRB2: leukocyte immunoglobulin-like receptor, subfamily B, member 2
POSH: plenty of SH3s
ROCK: Rho kinase
LZK: leucine zipper kinase (MAP3K12)
SD2: Shroom3 domain 2
R2C1: Shroom3 binding region of ROCK II
ELISA: enzyme linked immunosorbent assay
PPI: protein–protein interaction
GST: glutathione S-transferase
CGNs: cerebellar granule neurons.
